# Role of Digital Economy in Rebuilding and Sustaining the Space Governance Mechanisms

**DOI:** 10.3389/fpsyg.2021.828406

**Published:** 2022-01-21

**Authors:** Cen Cai, Ran Qiu, Yongqian Tu

**Affiliations:** ^1^School of Finance, Shanghai University of Finance and Economics, Shanghai, China; ^2^Chengdu Santai Intelligent Technology Co., Ltd., Chengdu, China; ^3^Research Fellow of National Academy of Development and Strategy, Renmin University of China, Beijing, China

**Keywords:** sustainable economic space, IT governance mechanism, corporate governance, ethics and awareness of employees, the environmental aspect

## Abstract

The need for sustainable corporate governance has gained the interests of researchers for a while now and it has been found as a very significant component of successful organizational operations. The current paper has examined the role of sustainable corporate governance in achieving sustainable economic space along with measuring the indirect impact of technological innovation and IT governance on the whole process. This paper has followed the quantitative-positivism approach to measure the hypotheses developed in the study. The population considered in this study are the managers currently employed in the corporate sector in China (*N* = 310). The data is analyzed using the Smart-PLS 3.3.3 software for checking the data for preliminary screening and the measurement of hypotheses. The findings of the study show that the three components of sustainable corporate governance, i.e., concern for employees, sustainable corporate governance awareness, and the environmental aspects have a significant as well as a positive effect on technological innovation and hence on the sustainable economic space. Similarly, the study has recorded a significant moderating effect of IT governance on the relationship of technological innovation and the sustainable economic space. Overall, it can be seen that corporate governance, innovative technology, and a sustainable digital economy share a reciprocal relationship. Where corporate governance helps as a supporting force to keep both innovation and sustainability in action. Whereas IT governance provides enhanced communication and delivery of public services, business, and advanced human capital. The study will be of high advantage for the corporate sector in China for devising and modifying their policies that consider the employee’s concerns for the governance mechanisms prevailing at priority in the organizations. Further, it will be interesting for the organizations to incorporate the IT governance mechanism in their technological innovations for achieving a sustainable economic space.

## Introduction

Today, the digital economy offers a diverse range of services that have impacted various traditional economic sectors including banking, transportation, health, education, publishing, and energy. This is because information and communication technologies have changed the dynamics of business as well as personal interactions. The digitally empowered platforms are the major catalyst of economic growth worldwide (Alam et al., 2018). Surprisingly, recent research studies have proved that the most significant feature of the digital economy is not technology but sustainable innovation. In this regard, the internet has provided ample opportunities for creative minds to invent and develop new solutions for old problems related to sustainability. The e-businesses unlike traditional business sectors such as oil and energy sectors address all the major environmental and social concerns on the very basic levels. The digital business models fully comprehend the seriousness of the issue of environmental sustainability rather than postponing them for the near future when it can endanger economic as well as environmental growth. Therefore, it is easy for the small enterprises which are young to implement new sustainability measures more conveniently than traditional business organizations stuck with old mindsets. Hence, with the right vision, smart policies and innovative imagination the digital economy can help to achieve both environmental and social sustainability (Miller and Wilsdon, 2001).

Furthermore, the advent of digital innovation has given birth to multiple social and economic changes (Oliveira et al., 2020). The traditional economy has faced a complete shift into a digitalized one. This digital transformation is highly dependent on the rate of innovation in data and technology (Bukht and Heeks, 2017). Unlike past, digital innovation is no more a top priority of software houses only but today every business organization needs this specific feature to become a part of a sustainable economy (Ciriello et al., 2018). Since the digital economy is complex in its dynamics it is a challenging task to acquire constant innovation (Fichman et al., 2014). These challenges include building transformed and updated business models that are more specific to small enterprises as well as medium enterprises (Scuotto et al., 2019). The growth of small enterprises is dependent on the strategic plan devised by SMEs to tackle these challenges. All organizations operating within the digital economy require a constant and high level of digital innovation in order to improve their technical skillsets. Moreover, the structural changes within any business organization are required to be analyzed in order to understand sustainability (Nguyen et al., 2015).

Generally, digital innovation is referred to as a constant process of improvement or a change any product or a business goes through to achieve sustainability in the digital economy (Ciriello et al., 2018). The current digital economy requires a constant innovation that is sustainable enough to support a further organic, green, and long-lasting future in a stable setting. The focus of the majority of existing models of digital innovation is on performances, revenue, process, marketing, and consumers. On the other hand, a sustainable model also addresses environmental as well as social factors such as employee concerns and preserving the natural resources. The sustainable model of innovation is nature inclusive and not only inspired by technology, but it also protects the environment and considers the social goals and not only commercial and financial goals. The concept of sustainable innovation has spiked after the 4th industrial revolution that is known by the commercialization of inventions such as robotics, the internet of things, 3D printing, cloud computing, autonomous vehicles, etc. (Avotra et al., 2021b; Nawaz et al., 2021; Yingfei et al., 2021).

The process of sustainable innovation is aided by both digital technology and management skills to bring forward creative business procedures and innovative solutions (Zairis, 2021). For a similar reason, today small enterprises usually adopt new technologies, software, or digital platforms to perform their regular functions in order to match the pace of the growing digital economy. Sustainable digital innovation in small or big businesses is supported by the internet of things and the gradual digital transformation. This innovation is different for different digital platforms based on their reach, features, management framework. Therefore, any business can alter these digital platforms according to their requirements to achieve sustainable digital innovation.

The current major product of digital innovation is the birth of e-commerce that has shifted the power dynamics between customers and organizations. Today the clients have many major benefits including being able to compare the prices of different goods with just one click. Although, it is to be noted that customers aren’t the sole stakeholders that take advantage of the e-commerce structure almost every stakeholder involved in the process can get benefited. However, whereas the internet is undoubtedly an inclusive tool with responsible economic models and it has helped the economy to grow. It still needs to formulate ethical and lawful boundaries for higher sustainability and security. The frequent issues of privacy and security jeopardize the reliability of the online markets and push customers to have reservations regarding online business transactions. With the advancement of digital technology, a more sophisticated and safer model of interaction for both businesses and customers is required. This sustainable and secure model can help businesses to avoid the multiple ranges of issues including the social and environmental impact of their products (Miller and Wilsdon, 2001). Numerous countries around the world have focused their attention on achieving sustainable economic spaces. The sustainability of the digital economy depends on a very important factor “good corporate governance.” Good governance is not only required for social and political spaces but also economic spaces. Especially in the case of the digital economy good corporate governance can resolve customers’ trust issues regarding the security of online transactions. Improved faith and confidence of the public can promote a digital economy offering sustainability for both environment and social fabrics (Durnev and Kim, 2005). Hermalin (2005) defines corporate governance as an environment of confidence, mutual trust, morals, ethics, and values among all the stakeholders of society including the public, professionals, businesses, corporate sectors, and government. Under this governance, every organization realizes that each action has a certain consequence that is the mutual concern of all social stakeholders now. Due to its popularity worldwide, corporate governance has gained significant importance in the last few years. The two major reasons for the spiked interest of the world in corporate governance are the economic sustainability and decentralization of business and industry as well as the demand for innovative and clear ethical boundaries along with strict law and order for corporate sectors (Joyner and Payne, 2002). Bushman and Smith (2001) have also pointed out another factor responsible for the sudden upsurge of awareness towards the new paradigm of corporate governance which is the existing demands of consumers for increased accountability and transparency from the companies.

Considering the importance of a sustainable digital economy, a large number of countries have implemented IT governments or e-governments by digitalizing the public services (Nograšek and Vintar, 2014). IT government as a procedure used by the government departments and other authorities to develop business and service delivery by using information and communication technologies (ICTs). The corporate IT governments can help the digital economies of developing countries to solve public services-related issues and improve the overall governance framework and management. There can be multiple advantages of IT governance such as accountability, decreased corruption, higher level of revenue production, reduction in managerial costs, and mainly enhanced transparency. IT government has the capacity to guide both private and public sectors to devise policies to improve their services and platforms. Khan and Vorley (2017) have cited a study that explains the IT governance has the ability to evaluate the policies and enact laws. This can lead to an overall increase in transparency and accountability that can support mitigating poverty and achieving a sustainable economy. Researchers and theorists of management sciences have already recognized the importance of quality and convenience of interactions as well as the significance of sharing as a major part of a sustainable society. Given the context, the IT government can play a major role in the transformation of society as well as the economy into a more sustainable one (Hao et al., 2020). Hence, both the digital economy and the IT government have a correlated impact on the sustainability of economic growth across the globe.

Meanwhile, due to digitalization the traditional boundaries among almost all the sectors are distorted. The world of the internet is focused on eradicating the class differences in the accessibility of information and public services provided by governments. The current paper aims to explore this multifaced relation between digital economy, sustainability, and the role of good corporate governance in the whole process. It is significant to measure this relationship since the research and development in this sector are important to achieve a unified model for sustainable economic spaces. The study also explores the impact of digital innovation due to its proven significance in achieving sustainable economic spaces. In nutshell, the study acknowledges that with a fast-growing digital economy, it’s almost impossible for a business to achieve a constant process of innovation that is both socially and environmentally sustainable. Hence, sustainable economic growth can only be achieved by regulating these economic spaces under the proper models of corporate governance. The current study aims to explore the effect of corporate governance that is further divided into three different factors on the innovative technologies in order to achieve the sustainable economic spaces.

## Review of Literature

There is ample of theoretical evidence that acknowledges the significance of examining the link between the sustainable digital economy and corporate government, empowered through technology also known as e-government. It would be important to investigate this relation to understand the social, political, demographic, legal, and economic factors that mediate the relationship between corporate governance and a sustainable digital economy. Numerous studies have already examined the development of the relationship between corporate government and sustainable digital economy (Bekkers and Homburg, 2009; Andersen et al., 2010). The literature review (Heeks and Bailur, 2007; Yildiz, 2007) dealing with the examination of different e-government models, has found that most of the models are built on weak theoretical foundations. The studies argue that there is a clear need for further insights and evidence to comprehend the concept of good governance for sustainable economic spaces. Moreover, Shareef et al. (2011) have observed that discussions on this issue are dispersed. Researchers have also observed that most of these e-government models are only supported by discussions and there is an absence of scientific evidence to support the utility of given models. Another study Zhao et al. (2015) in a similar context has examined the relationship between the digital economy and corporate government at the global level by examining 67 countries including both developed and underdeveloped.

### Sustainability

During the last quarter of this century, multiple researchers have acknowledged the fact that actions of any organization can have both internal as well as external consequences. Therefore, an organization should be conscious as well as accountable for its actions to the larger audience instead of only to its stakeholders. This sort of recommendation was first recorded in 1970. Similarly, many philosophers also highlighted the role of business as a member of society as well as the significance of its social performance. The businesses were aware of the requirement to adapt to community climate and be accountable but their constant focus on revenue generation had hindered the social receptiveness. Similar ideas were proposed by McDonald and Puxty (1979) who recognize companies as a social tool and not only properties of stakeholders, hence, called for the need for greater accountability. Subsequently, Rubenstein (1992) highlighted the need for a new social contract between businesses and their stakeholders. This social contract is centered on the concern for the future through the concept of sustainability. The term sustainability has been largely popular in both global and as well as corporate discourse. For the same reasons, it has been defined in several ways. Crowther (2016) has given the broadest definition of sustainability that is focused on the availability of the options in the future for the effect of any action taken in present. In short, the use of resources in the present should be careful to save them for the future as the earth has limited resources. Hence, Visser and Hawken (2013) recommend there shouldn’t be more resources used than regenerated. The capacity of the ecosystem as well as both input and output models of resource consumption should be under constant consideration. Therefore, sustainability views an organization in the wider social and economic context not just for revenue generation or value creation but also for the future of the business as well as society itself (Avotra et al., 2021b).

The concept of corporate sustainability has become conflicted since the time it has emerged. There are two common assumptions. First that it refers to development that is sustainable in its nature. The second assumption is that to achieve sustainability companies are only required to acknowledge social and environmental issues and make them part of their strategic plans. However, Werre and van Marrewijk (2003) believe that there is no single definition of sustainability but every company requires to formulate its own concept of sustainability under the framework of its goals and objectives. Most of the explanations of sustainability have not acknowledged financial performance as an important part of the concept (Yingfei et al., 2021).

### Sustainability and Innovative Technology

Today most companies are required to adapt to digital as well as innovative technologies in order to support their survival in a sustainable economy while also working for social welfare by answering the concerns of the public (Klein, 2020). The digital transformation can enable any business to avail the wider range of opportunities related to value creation as well as becoming an integral part of the digital economy (Kane et al., 2015). However, the digital transformation cannot stay sustainable unless a business is ready to adopt updated and innovative technology-based solutions (Garcia De Lomana et al., 2019). The potential of an organization to adopt innovative technologies depends on the ability of any organization to use digital activities in its daily operations. Innovative technologies can lead to positive change in work environments and gradually the entire business context (Khin and Ho, 2019). To achieve sustainable digital innovation organization needs to show high interest in optimization, client interactions, and adapting to digital technologies (Bican and Brem, 2020). Sustainable digital innovation is not only dependent on the relationship between digital platforms and technologies but is also impacted by the extent of influence an organization’s digital transformation has on the speed of digital innovation.

In order to achieve sustainability both digital innovation and economic spaces require regulatory frameworks (Zhao et al., 2015). For this many countries have acquired digital services to introduce the IT governments. In this regard, United Nations has set a dynamic approach to evaluating the quality and development of IT governments implemented in its member states. This approach helps to analyze the standard and value of public, educational, economical, and health services delivered by the IT governments. The same approach has helped to evaluate the e-participation of different countries from three different levels; the transmission of information, discussion, and participation of citizens and governments in the process of decision making. Through this strategy, the United Nations has highlighted the significance of good corporate governance. Moreover, research also shows that the IT government does mediate the relationship between a sustainable digital economy and digital innovation (Lee and Berente, 2012). Hence, given the importance of both IT governance and innovation technology in achieving sustainable economic spaces, the current research aims to explore the mediating effect of IT governance on the direct relation between sustainable economic spaces and digital innovation. This can be formulated into the following hypotheses.

H_1_: *Innovative technology has an association with sustainable economic performance.*
H_2_: *IT governance moderates the relationship of innovation technology and sustainable economic performance.*

### Corporate Governance

The debate about sustainable digital Innovation cannot be carried alone without discussing the next big issue of corporate governance acknowledged globally. Many companies aim to become global and at the same time, they want to achieve sustainability to keep the competitive advantage. The concept of corporate government started to attract attention at the global level in the mid-1980s. Today, almost all professionals including managers, government officials, auditors, and human resource organizations have recognized its need as well as importance. The code of good governance by Anglo-Americans offered the right amount of motivation to develop the concept further. These codes have been adopted by the economies of both first worlds as well as developing countries into their own set of principles and goals. This set of goals has been chosen above legal standards mostly. With increasing risks for investors, corporate governance has become a safe tool since it fulfills the demand of stakeholders to implement strict corporate principles to guide the businesses. For this purpose, investors have shown interest to invest more to promote the principal implementation of corporate governance (Beiner et al., 2006). In today’s digital economy corporate governance reports are the major tools to attract the interest of investors to secure funding for sustainable ventures. The other reason for encouraging corporate governance is that it helps to deal with the risk involving banking and credit scams. It also offers solutions for devising new rules to guide companies financial evaluations. Apart from as a tool for risk measurement, corporate governance helps to establish better credibility of the company. For example, if a company requires a high score in the rating process, then it has to formulate high-quality corporate governance rules. The rating agencies pay special attention to the standards of corporate governance along with a few other corporate indicators of growth. Apart from this, good corporate governance has become essential for stakeholders, auditors, and governments. For the same reason, corporate governance attracts the major attention of financial institutions, legal bodies, policymakers, researchers, and academics. This is one of the major explanatory factors that the connection between corporate governance and its actual performance is still under debate. There has been a significant number of research studies in the past dealing with examining the relationship between performance and corporate governance (Coles et al., 2001; Heracleous, 2001; Becht et al., 2002; Demsetz and Villalonga, 2002; Gompers et al., 2007; Bhagat and Jefferis, 2018). The results from the above studies have shown mixed and vague findings, without offering a clear status of the relationship. However, according to these studies, it seems that corporate governance is significant for better company performance, value generation, and reliability. According to Crowther (2007), there are mainly four principles of good corporate governance that are found in practice as well; accountability, fairness, transparency, and responsibility. It is to be noted that all four principles are related to the company’s social responsibility. According to Sethi (2002), corporate governance is all about attaining the balance between economic and social goals including accountability and efficient use of resources as well as the behavior of a company towards its employees. Therefore, addressing the social factors of corporate governance supports sustainability in economic spaces.

### Sustainable Corporate Governance and Technological Innovation

Good corporate governance has been under the discussion for many years, to attain a single clear definition of the concept. However, according to Gray et al. (2001), there are a few distinct characteristics that can be found in any functional model of good governance. These characteristics include creating sustainable value, attaining the balance between the economic and social advantages as well as the company’s set goals. Good corporate governance provides long-lasting benefits related to risk mitigation, attracting more investments and stakeholders. There have been studies in the past exploring the benefits of corporate governance (Cowen et al., 1987; Burke and Logsdon, 1996; Gray et al., 2001). The results of these studies show that these benefits are dependent on the sustainability of the business. Subsequently, these research studies guide to pay attention to the concept of sustainable corporate governance within a business organization. Despite the clear link established by previous studies between good governance and sustainability, it should be noted that these definitions are specific to a particular context since the concepts are defined by companies. Most of these companies have their own opinion about good corporate governance and sustainability and do not understand the relationship between these two. Although there is a clear link between good corporate governance and all aspects of the performance of any organization. Hence, researchers have not settled upon any particular aspect of governance. Instead, they have accepted the firms’ definitions of the concept and have focused their attention on what they say about governance and its relationship to sustainability.

Since the literature review has already established that corporate government encompasses many factors that directly affect the sustainability of a business. The previous research shows that this relationship is not at all clearly understood by many firms. Before understanding good corporate governance, it is important to understand the four major aspects of sustainability presented by Crowther (2007) that must be the part of good corporate governance;

1) *Social Impact:* It is the measure of the influence of society and stakeholders on the company including both ethical and corporate sets of rules or principles.
2) *Environmental Influence:* This aspect of sustainability deals with the measurement of the effect of a company’s actions on the geographical and physical fabric surrounding the environment.
3) *Organizational values:* This factor is entirely related to the company’s relationship with its internal stakeholder specifically employees.
4) *Financial Impact:* This measures the decent return for the levels and extent of risks taken by a company.

These are four major key factors to achieve sustainability in corporate governance. As they capture both internal and external factors, long-term and short-term goals in the context of both present and future. These factors embedded in any model of corporate governance pave a path towards sustainability. Subsequently, this sustainability offers the regulated distribution of both negative and positive effects and helps to remove the conflict between the two as well. Hence, the absence of sustainability in corporate governance leads towards the traditional short-term approach that is no more feasible in the always-changing digital economy (Werre and van Marrewijk, 2003). Therefore, it is a need of time to conduct a detailed investigation on the influence of different factors of sustainability (concern for employees, environmental concern, and sustainable corporate governance) embedded in a corporate government on the innovative technologies and their role in bringing sustainability to the digital economy. It enables the following hypotheses.

H_3_: *Concern for employees has an association with innovative technology.*
H_4_: *Ethical and corporate governance awareness has an association with innovation technology.*
H_5_: *The consideration for environmental aspect has an association with innovation technology.*

### Technological Innovation and Sustainable Digital Economy

It is observed that the existing post-industrial social fabric demands an economy based on knowledge and sustained by innovative technology as well as smart human capital resources (Zheng et al., 2020). The research study provides insights into the major lags between the needs of an individual and a group. However, the gap is identified and dealt with through the emergence of smart cities and the internet of things. The existing structure of the digital economy is going to alter the nature of innovative technologies including recreating new types of social and organizational reforms related to employees, the environment, and ethics. Dozens of American, European, and Asian countries are already focusing on developing economic spaces that can sustain through good corporate governance and are supported through innovative technologies (Chai, 2019). These countries are leading in the development of sustainable economies through constant digital innovation related to human resources, science, technology, and research and development. These innovations are important to achieve sustainability as well as require a strict corporate governance framework to guide them. However, on the other side digital innovation has been proven to be a destruction for the traditional business models but also a major catalyst for a sustainable digital economy. Organizations are rethinking their priorities and are stimulated to redesign their existing organizational models into more inclusive and digitalized organizational environments. This would lead to the overall sustainability of the economic spaces through innovative technology working under the good corporate governance model (Yun et al., 2020). Lastly, the digitalization of organizational environment can be achieved through innovative technologies that can revolutionize social interactions and human relationships through a more socially inclusive environment and offer integrated communication opportunities. However, the other side of this same picture is also discussed by Goos et al. (2014) who have pointed out some disadvantages of these innovative technologies. For instance, digital platforms have disrupted the labor forces, provided an opportunity for internet scams and cybercrimes, the disappearance of companies, and social loneliness at discrete levels. These few challenges push the policy and governance regulators to comprehend the process of innovation more clearly. It reveals that digital innovation is an ever-changing process of transformation. Corporate governments must understand these changing patterns so the information and communication technology officials and policy regulators can foresee the fluctuations caused by the future waves of change, leading to better implementation of sustainability and corporate governance.

Previous studies have only highlighted a few factors including strategies related to digital innovation, sustainability, organizational perspective, environmental impact, division of innovative employees, digital workplace as well as organizational readiness and learning (Sambamurthy et al., 2003; Yoo et al., 2005; Lin, 2008; Lee and Berente, 2012; Nylén and Holmström, 2015; Demirkan et al., 2016; Shahzad et al., 2017; Hinings et al., 2018; Huesig and Endres, 2019; Lokuge et al., 2019). A comprehensive study is required to examine the influence of corporate governance in achieving a sustainable economy under the direct influence of innovative technologies and mediating influence of IT governances. The current study explores the influence of innovative technologies on the factors involved in the establishment of corporate governance. Moreover, it examines the impact of corporate governance on innovative technologies as well as attaining sustainable economic spaces through digitalization. To achieve a sustainable economy the digitization should be understood as two progressions at once including constant development through innovative technologies and research and expansion. Therefore, under the findings of previous studies by Fountain (2002), Sang et al. (2009), and Hung et al. (2013), there is a diverse range of roles including employees, policymakers, environmental, educational, and political factors that influence the sustainability of corporate governance and digital innovation that has a further direct impact on the sustainability of economic spaces. Hence the current study hypothesizes as:

H_6_: *Innovation technology has a mediating effect on the relationship of concern for employees and sustainable economic space.*
H_7_: *Innovation technology has a mediating effect on the relationship of ethical and corporate governance and sustainable economic space.*
H_8_: *Innovation technology has a mediating effect on the relationship of consideration for environmental aspect and sustainable economic space.*

**FIGURE 1:**
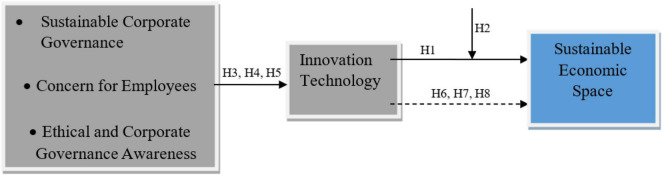
Conceptual model.

A following conceptual model (Figure 1) has been formed based on the above literature and hypothesis.

## Methodology

The current study follows a quantitative methodology with the deductive approach where hypotheses were developed and the study was checked for the effect of certain variables on other variables. This methodology has been used for the minimum bias. The data in this quantitative study has been collected through the self-administered survey method. The population taken for this study is the managerial staff of corporate organizations. The sample selection method used in the current study is convenience sampling, since reaching out to each manager is not easy, though reaching those given consents for being part of the survey was convenient. Therefore, a convenient sampling design was used. The questionnaires were distributed and collected a week later. The usable questionnaires received were 310 and they were used for data analysis. The unit of analysis in this study was the managers of the corporate sector in China.

### Instrument Development

The survey instrument used in this study was the questionnaires. The questionnaire consisted of questions related to each variable. There were six variables in total for this study one independent variable, i.e., sustainable corporate governance that was split into three sub-variables (i.e., concern for employees, ethical and corporate governance awareness, and environmental aspect), one dependent variable i.e., sustainable economic space, one mediating variable, i.e., IT innovation and one moderator, i.e., IT governance mechanism. All the scales were adapted from the previous studies, e.g., the scale for sustainable corporate governance was adapted from Rampasso et al. (2019), IT innovation from Clauss (2016), IT governance mechanism from Kuruwitaarachchi (2020), and the dependent variable of sustainable economic space from Calik and Bardudeen (2016). The adapted questionnaire followed the five-point Likert scale for responses of the respondents, where 1 was given for the strong disagreement and 5 for a strong agreement. The concern of employees was measured with five items, ethical and corporate governance awareness with five items, and environmental aspect with four items. The mediating variable innovation technology was measured with three items, the dependent variable of sustainable economic space with four items, and the moderating variable IT governance mechanism with three items as well.

### Demographics Details

The data analysis done in this study in three stages. In the first stage, the demographics of the respondents were analyzed using frequency and percentages. The differences were noted on the basis of gender (55.16% males and 44.83% females), age with highest contribution from the age segments of (15–20) and (21–25), education with three categories of bachelors, masters and Ph.D. and others; and the nature of job. The details have been mentioned in the Table 1.

**TABLE 1:** Demographics analysis.

Demographics	Frequency	Percentage %
**Gender**		
Male	171	55.16
Female	139	44.83
**Age**		
15–20	103	33.22
21–25	110	35.48
26–30	72	23.22
31 and above	25	8.06
**Education**		
Bachelors	142	45.80
Masters	115	37.09
Ph.D. and others	53	17.09
**Nature of Job**		
Permanent	142	45.80
Contract	168	54.19

*N = 310.*

## Data Analysis and Results

Data of the study were analyzed using the software used widely for structural equation modeling (SEM), i.e., Smart PLS 3.3.3. Using this software, the data is analyzed in two stages; the first is the measurement model and the second is the structural model. In the measurement model, the validity and reliability of the data are checked. For validity, factor loadings of the items, average variance extracted, hetero trait mono trait ratio, and Fornell and Larcker tests were used. On the other hand for reliability, Cronbach Alpha reliability and composite reliabilities are used.

### Measurement Model

**FIGURE 2:**
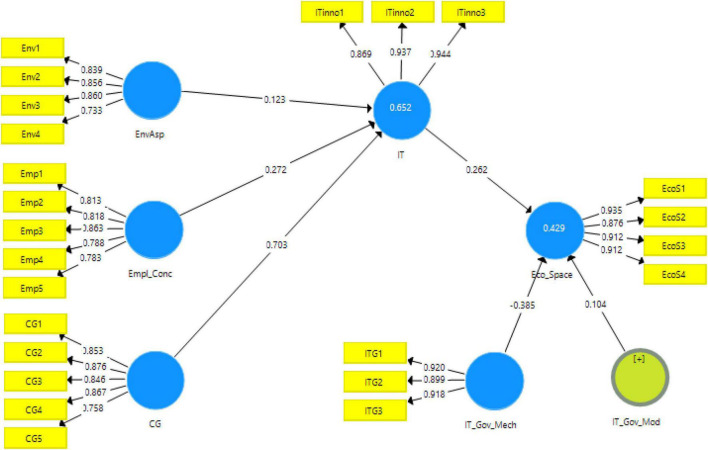
Output of measurement model algorithm. EnvAsp, environmental aspect; Empl_Conc, concern for employees; CG, ethical and corporate governance awareness; Eco_Space, sustainable economic space; IT, technological innovation; IT_Gov_Mech, IT governance mechanism.

The measurement model algorithm obtained is shown in Figure 2. It shows the contribution of the independent variables in dependent variables.

The statistics obtained for factor loadings of each variable, alpha reliability and composite reliability along with AVE have been reported in Table 2. It shows that the factor loadings are all above the threshold mentioned in the literature, i.e., 0.7 (Nawaz et al., 2020). The variance inflation factor is also mentioned that are under the prescribed ranges of less than 2.5 is medium while up to 4 is acceptable (Sarstedt et al., 2019). These values are given in Table 2.

**TABLE 2:** Factor loadings and VIF.

Variables	Factor Loadings		VIF
Environmental aspect	EA 1	0.839	1.942
	EA 2	0.856	2.171
	EA 3	0.860	2.119
	EA 4	0.733	1.577
Employee concern	EC1	0.813	2.467
	EC 2	0.818	2.105
	EC 3	0.863	3.521
	EC 4	0.788	2.380
	EC 5	0.783	1.541
Ethical and corporate governance awareness	CG1	0.853	2.460
	CG2	0.876	2.795
	CG3	0.846	2.409
	CG4	0.867	2.613
	CG5	0.758	1.684
Technological innovation	TI1	0.869	1.990
	TI 2	0.937	2.763
	TI 3	0.944	2.035
IT governance mechanism	CS1	0.920	2.939
	CS2	0.899	2.534
	CS3	0.918	2.968
Sustainable economic space	SE1	0.935	2.175
	SE2	0.876	2.674
	SE3	0.912	3.521
	SE4	0.912	3.608
	SE5	0.935	2.175

Similarly, the cutoff point of Cronbach alpha reliability is also 0.7 (Hair et al., 2017). All the values in this study for Cronbach alpha and composite reliability are above 0.7 ranged from 0.842 to 0.930 for Cronbach alpha. Similarly, the AVE for the variables of the study ranged from 0.622 to 0.841 as shown in Table 3.

**TABLE 3:** Alpha reliabilities and AVE.

	Cronbach alpha	Composite reliability	AVE
Environmental aspect	0.842	0.894	
Employee concern	0.874	0.907	0.662
Ethical and corporate governance awareness	0.896	0.923	0.708
Technological innovation	0.905	0.941	0.841
IT governance mechanism	0.899	0.937	0.832
Sustainable economic space	0.930	0.950	0.826

Discriminant validity of the variables in the current study has been checked through the most commonly used tests namely Hetero trait Mono trait Ratio and Fornell and Larcker Criteria. The results for these two tests are reported in Tables 4, 5. The heterotrait-monotrait ratio of correlations (HTMT) ratios are said to be below 0.9 for to data be validated (Franke and Sarstedt, 2019). All values in Table 3 are below 0.9 thus verifying the HTMT ratio significance in the present study. In the same way, the Fornell and Larcker criteria also showed the highest values at the top in each column. The results for Fornell and Larcker criteria can be seen in the Table 4.

**TABLE 4:** HTMT ratio.

	CG	Eco_ Space	Empl_ Conc	EnvAsp	IT	IT_Gov _Mech
CG	–					
Eco_Space	0.622					
Empl_Conc	0.605	0.399				
EnvAsp	0.640	0.341	0.802			
IT	0.870	0.620	0.615	0.519		
IT_Gov_Mech	0.792	0.645	0.450	0.538	0.680	–

*EnvAsp, environmental aspect; Empl_Conc, concern for employees; CG, ethical and corporate governance awareness; Eco_Space, sustainable economic space; IT, technology innovation; IT_Gov_Mech, IT governance mechanism; HTMT, heterotrait-monotrait ratio of correlations.*

**TABLE 5:** Fornell and Larcker criteria.

	CG	EcoSpace	EmplConc	EnvAsp	IT	ITGovMech
CG	0.841					
Eco_Space	0.569	0.909				
Empl_Conc	0.553	0.376	0.814			
EnvAsp	–0.560	–0.308	–0.686	0.824		
IT	0.785	0.571	0.576	–0.457	0.917	
IT_Gov_Mech	–0.711	–0.591	–0.410	0.475	–0.614	0.912

*EnvAsp, environmental aspect; Empl_Conc, concern for employees; CG, ethical and corporate governance awareness; Eco_Space, sustainable economic space; IT, technology innovation; IT_Gov_Mech, IT governance mechanism.*

Furthermore, the *r*-square values for the mediating variable technological innovation are 65.2% and that of the dependent variable sustainable economic space is 42.9%.

### Structural Model

**FIGURE 3:**
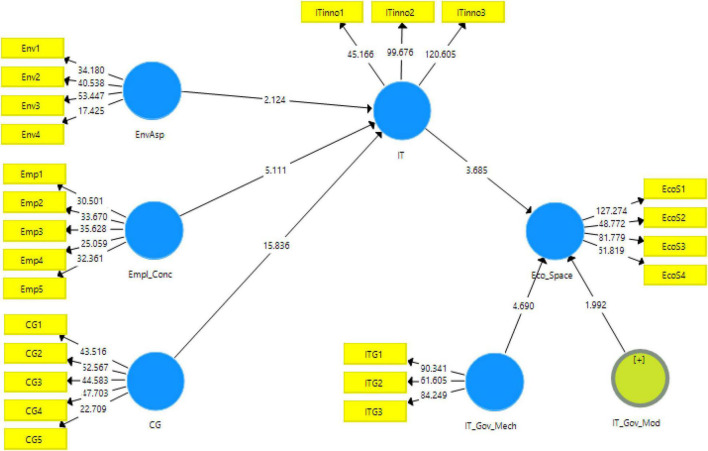
Output of structural model algorithm. EnvAsp, environmental aspect; Empl_Conc, concern for employees; CG, ethical and corporate governance awareness; Eco_Space, sustainable economic space; IT, technological innovation; IT_Gov_Mech, IT governance mechanism.

The structural model algorithm obtained from the Smart PLS is shown in Figure 3. According to the results obtained, the hypotheses are either accepted or rejected based on the statistics obtained in this model. The key acceptance criteria used in the present study are *t*-statistic, *p*-values, the original sample mean values, and standard deviation values. The details of these statistics are reported in Table 5.

The structural model gives the output regarding the key indicators for accepting or rejecting the proposed variables. In this study, the direct effects of the variables have been shown in Table 6.

**TABLE 6:** The direct effects of the variable.

Paths	*H*	*O*	*M*	*SD*	*T*-statistic	*P*-value	Results
Emp_Con → IT	H_1_	0.272	0.272	0.053	5.111	0.000***	*Accepted*
CG → IT	H_2_	0.703	0.701	0.044	15.836	0.000***	*Accepted*
ENV_Asp → IT	H_3_	0.123	0.117	0.058	2.124	0.034*	*Accepted*
IT → Eco_S	H_4_	0.262	0.257	0.071	3.685	0.000***	*Accepted*

***p < 0.001, *p < 0.05.

*H, hypothesis; O, original sample; M, sample mean; SD, standard deviation; EnvAsp, environmental aspect; Empl_Conc, concern for employees; CG, ethical and corporate governance awareness; Eco_Space, sustainable economic space; IT, technological innovation; IT_Gov_Mech, IT governance mechanism.*

In the first hypothesis of the study, concern for employees has shown a significant and positive effect on technological innovation with *t*-statistic = 5.111 and *p*-value <0.000. Similarly, the second and fourth hypotheses also showed significant positive results for corporate governance effect on technological innovation (H_2_: *t*-statistic = 15.836 and *p*-value = 0.000 accepted at *p*-value <0.001) and its effect on sustainable economic space (H_4_: *t*-statistic = 3.685, *p*-value <0.000). While, H_3_ has also been accepted at *t*-statistic = 2.124 and *p*-value = 0.05.

Moving to the indirect effects, the moderating effect of IT governance mechanism has been found significant in the relationship of technological innovation and sustainable economic space with *t*-statistic = 1.992 and *p*-value less than 0.05. Similarly, the hypotheses H_6_ (*t*-statistic = 2.805, *p*-value = 0.005) and H_7_ (*t*-statistic = 3.641, *p*-value = 0.000) have also been accepted, however, hypothesis H_8_ could not find any significance in the study as depicted in Table 7.

**TABLE 7:** The indirect effects of the variable.

Paths	*H*	*O*	*M*	*SD*	*T*-statistic	*P*-value	Results
IT_Gov_Mech _Mod → Eco_S	H_5_	0.104	0.111	0.052	1.992	0.047*	*Accepted*
Emp_Con → IT → Eco_S	H_6_	0.071	0.070	0.025	2.805	0.005*	*Accepted*
CG → IT → Eco_S	H_7_	0.185	0.180	0.051	3.641	0.000***	*Accepted*
ENV_Asp → IT → Eco_S	H_8_	0.032	0.030	0.018	1.776	0.076	Rejected

***p < 0.001, *p < 0.05.

*H, hypothesis; O, original sample; M, sample mean; SD, standard deviation; EnvAsp, environmental aspect; Empl_Conc, concern for employees; CG, ethical and corporate governance awareness; Eco_Space, sustainable economic space; IT, technological innovation; IT_Gov_Mech, IT governance mechanism.*

## Discussion

The research has explored the direct effect of factors related to corporate governance (concern for employees, environmental concern, and sustainable corporate governance) on the innovative technologies. In addition, it examines the mediating role of an innovative technology on sustainable economic spaces and moderating role of IT governance during this process. The results from *t*-statistic, *p*-values, the original sample mean values, and standard deviation values have revealed that concern for employees has shown a significant and positive effect on innovation technology with *t*-statistic = 5.111 and *p*-value <0.000. It indicates the need for smart and innovative human capital for sustainable innovation (Yuan and Woodman, 2010). Similarly, there is a positive effect of sustainable corporate governance on the innovative technology which further has a significant effect on a sustainable economic space. This reveals a triangular relationship between the three and the fact that sustainability in the economy cannot be achieved through only innovation but it also requires a set of clear principles and rules to guide the whole process in any economic space (Yousaf et al., 2021). The triangular effect of innovative technology has been further proved as results show that it has a mediating effect on concern for employees, ethical and corporate governance as well as sustainable economic spaces. This reiterates the significance of innovative technology in achieving both sustainable corporate governance and the economy (Abraham et al., 2019). Moreover, the study has also found that consideration for environmental aspects has an association with innovation technology. This shows that innovative business models have to pay attention to generating green and sustainable solutions as well as important innovations for sustainable economic spaces (Avotra et al., 2021a). However, there has not been any statistical evidence found for the hypothesis stating innovation technology has a mediating effect on the relationship of consideration for environmental aspects and sustainable economic space. As far as the indirect effects are concerned it is found that the IT government has a significant mediating impact on the relationship between innovative technology and a sustainable economy. This has also been proved in various previous studies by Fountain (2002) who has explained that digital technologies are not applied but enacted under a proper set of principles devised by the policymakers. This highlights the importance of IT governance that can help to smoothly run the process of delivering business and public services while offering security and risk-free economy. For the same reason, IT governance has attracted attention worldwide especially in public administration sectors who have encouraged to adopt innovative technologies in public management (Gauld and Goldfinch, 2006). Overall, it can be seen that corporate governance, innovative technology, and a sustainable digital economy share a reciprocal relationship. Moreover, corporate governance helps as a supporting force to keep both innovation and sustainability in action. Whereas IT governance provides enhanced communication and delivery of public services, business, and advanced human capital. Subsequently, similar concepts of sustainability both in governance and corporate context are frequently analyzed by different national and organizations including the Organization for Economic Co-operation and Development (OECD) and the United Nations (UN) (Linkov et al., 2018). Hence the current study has examined the three major variables which are related to social, organizational, and environmental context. The results show all these three factors have a positive impact in achieving sustainable economic spaces as well as sustainable digital innovation. These results are significant to understand the power of knowledge management in evaluating the economic features as well as social factors (Foss, 2007). Hence, the application of corporate governance extended on all organizational, environmental, and social aspects can support the digital economy through innovative technology to achieve sustainable economic spaces.

## Conclusion

Corporate governance has been a vital component in the organizations and the impacts it forms on the environments around and the economy overall. The current study has also made such an attempt to find the role of corporate governance on the sustainable economic space by splitting it into three constituents of concern for employees, ethics and corporate governance awareness, and the environmental aspect. These three independent variables (i.e., concern for employees, ethics and corporate governance awareness, and environmental aspect) have found significant effects on technological innovation and sustainable economic space. The mediating effects checked with technological innovation have also been found to play a partially significant (i.e., concern for employees, ethics, and corporate governance awareness) role in predicting the effect of overall sustainable corporate governance on sustainable economic space for the organizations. Further, the moderating effect has also been found significant that the presence of IT governance mechanisms plays a vital role in using technological innovations for sustainable economic developments.

This study has certain implications associated with it for the corporate world. The study will be of high advantage for the corporate sector in China for devising and modifying their policies that consider the employee’s concerns for the governance mechanisms at priority in the organizations. Secondly, it will be interesting for the organizations to incorporate the IT governance mechanism in their technological innovations for achieving and contributing a sustainable economic space. Thirdly, it will lead the organizations in the right way using information technology mechanisms in their organizational procedures and code of conduct that will help the country attaining sustainable economic space at the mass level. However, there are certain limitations as well. This has used the population of managers in China, which can be checked in Europe and other Asian countries as well. Similarly, more indicators of corporate governance and sustainable economic space can be checked for their contribution for the technological innovation and digital progress.

## Data Availability Statement

The original contributions presented in the study are included in the article/supplementary material, further inquiries can be directed to the corresponding author/s.

## Author Contributions

RQ and YT conceived and designed the concept. CC collected the data and wrote the manuscript. All authors read and agreed to the published version of the manuscript.

## Conflict of Interest

RQ was employed by the company Chengdu Santai Intelligent Technology Co., Ltd. The remaining authors declare that the research was conducted in the absence of any commercial or financial relationships that could be construed as a potential conflict of interest.

## Publisher’s Note

All claims expressed in this article are solely those of the authors and do not necessarily represent those of their affiliated organizations, or those of the publisher, the editors and the reviewers. Any product that may be evaluated in this article, or claim that may be made by its manufacturer, is not guaranteed or endorsed by the publisher.
